# Impacts of Short-Rotation Early-Growing Season Prescribed Fire on a Ground Nesting Bird in the Central Hardwoods Region of North America

**DOI:** 10.1371/journal.pone.0147317

**Published:** 2016-01-21

**Authors:** H. Tyler Pittman, David G. Krementz

**Affiliations:** 1 Arkansas Cooperative Fish and Wildlife Research Unit, Department of Biological Sciences, University of Arkansas, Fayetteville, Arkansas, 72701, United States of America; 2 U.S. Geological Survey Arkansas Cooperative Fish and Wildlife Research Unit, Department of Biological Sciences, University of Arkansas, Fayetteville, Arkansas, 72701, United States of America; University of Sydney, AUSTRALIA

## Abstract

Landscape-scale short-rotation early-growing season prescribed fire, hereafter prescribed fire, in upland hardwood forests represents a recent shift in management strategies across eastern upland forests. Not only does this strategy depart from dormant season to growing season prescriptions, but the strategy also moves from stand-scale to landscape-scale implementation (>1,000 ha). This being so, agencies are making considerable commitments in terms of time and resources to this management strategy, but the effects on wildlife in upland forests, especially those dominated by hardwood canopy species, are relatively unknown. We initiated our study to assess whether this management strategy affects eastern wild turkey reproductive ecology on the Ozark-St. Francis National Forest. We marked 67 wild turkey hens with Global Positioning System (GPS) Platform Transmitting Terminals in 2012 and 2013 to document exposure to prescribed fire, and estimate daily nest survival, nest success, and nest-site selection. We estimated these reproductive parameters in forest units managed with prescribed fire (treated) and units absent of prescribed fire (untreated). Of 60 initial nest attempts monitored, none were destroyed or exposed to prescribed fire because a majority of fires occurred early than a majority of the nesting activity. We found nest success was greater in untreated units than treated units (36.4% versus 14.6%). We did not find any habitat characteristic differences between successful and unsuccessful nest-sites. We found that nest-site selection criteria differed between treated and untreated units. Visual concealment and woody ground cover were common selection criteria in both treated and untreated units. However, in treated units wild turkey selected nest-sites with fewer small shrubs (<5 cm ground diameter) and large trees (>20 cm DBH) but not in untreated units. In untreated units wild turkey selected nest-sites with more large shrubs (≥5cm ground diameter) but did not select for small shrubs or large trees. Our findings suggest that wild turkey have not benefited from the reintroduction of prescribed fire to the WRERA.

## Introduction

Over the past 40 years managers in eastern upland-hardwood public forests in North America have shifted from the use of dormant season prescribed fire to landscape-scale short-rotation early-growing season prescribed fire (hereafter prescribed fire) as a means to restore and maintain fire-tolerant oak-dominated forests, woodlands, and savannas [1–3]. During this transition period, numerous studies documented the effects that fire has on the flora and fauna in these ecosystems. For birds, prescribed fire in upland-hardwood forests either benefits or does not affect breeding birds, especially woodland generalists and those requiring early successional forests [4–8]. However, many research studies noted the potential adverse effects of prescribed fire on ground and low-shrub nesting birds [1,5–8].

**Fig 1:**
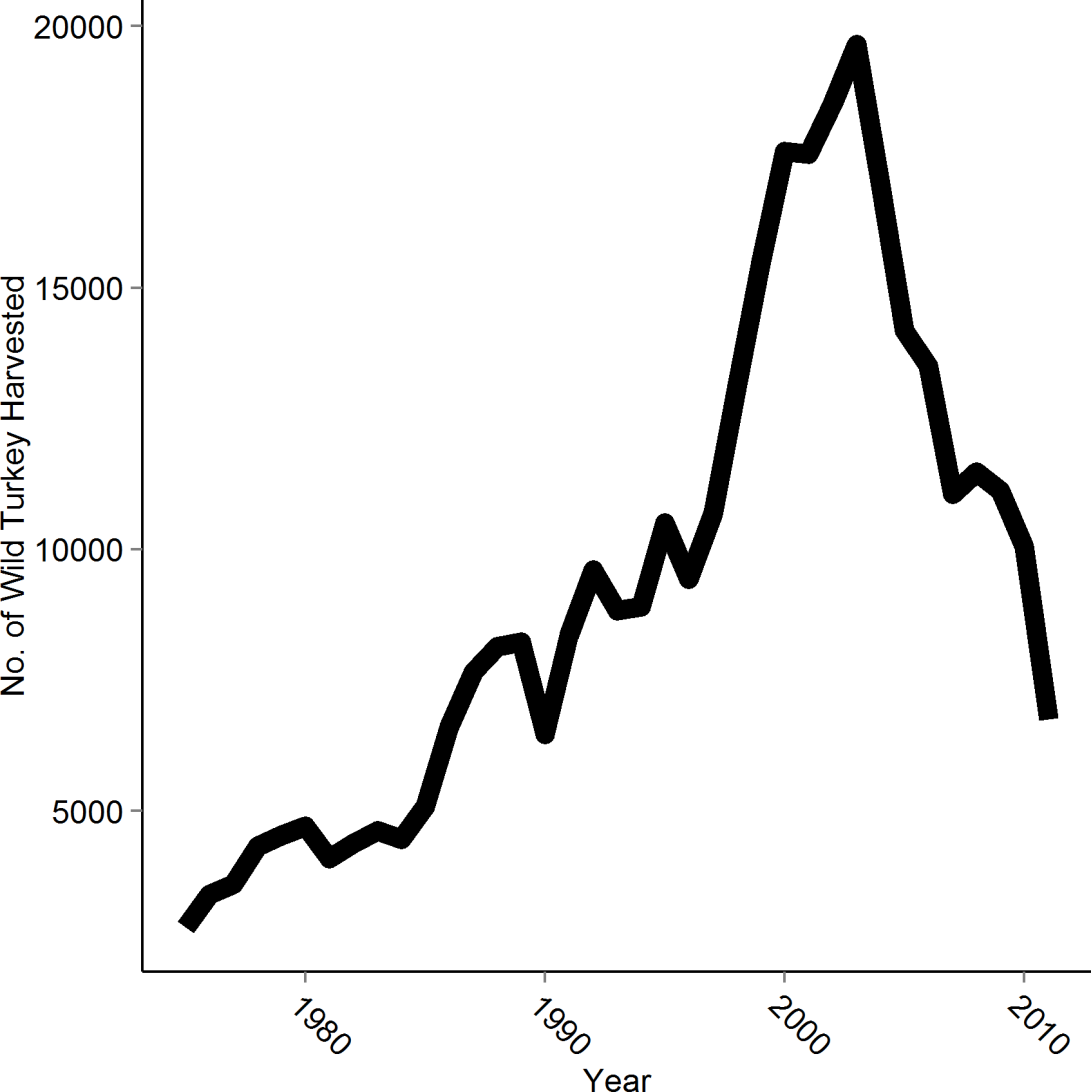
Estimated harvest of Eastern wild turkey (*Meleagris gallopavo silvestris*) in Arkansas from 1975 to 2010 [J. Honey, unpublished data, Arkansas Game & Fish Commission].

The Eastern wild turkey (*Meleagris gallopavo silvestris*) is a well-known and economically important ground-nesting bird that nests in upland-hardwood forests of Eastern North America. Since the early 2000s, harvest estimates of wild turkey in Arkansas and other southeastern states suggest a declining population (Fig 1) ([9]; J. Honey, unpublished data, Arkansas Game & Fish Commission). Since the onset of this possible decline, many studies have focused on the effects of management practices such as prescribed fire on wild turkey reproductive ecology. Unfortunately, none of the studies that have examined the effects of frequent prescribed fire on nest survival and nest-habitat selection for wild turkey have taken place in landscapes dominated by upland hardwood forests [10,11]. This neglect of upland hardwood ecosystems leaves a significant knowledge gap on how current forest management paradigms affect wild turkey and potentially other ground-nesting birds.

In addition, all recent studies of wild turkey and ground-nesting species (including those previously mentioned) were conducted on smaller scales where prescribed fire treatment units or patch sizes rarely exceeded 100 ha. Landscape-scale management is becoming more popular, especially on public lands as with the Collaborative Forest Landscape Restoration Program (CFLRP) of the United States Department of Agriculture (USDA) Forest Service. The goals of the CFLRP include, amongst others, to re-establish natural ecosystem processes in forested landscapes using forest management techniques that achieve ecological and watershed health objectives and make those ecosystems more resilient [12]. The success of the CFLRP implementation at many sites across the United States and other similar programs will depend on sound knowledge of the effects of large-scale prescribed fires (>1,000 ha) on wildlife.

Our objectives were to examine the effects of prescribed fire in upland-hardwood dominated landscapes on wild turkey nest success and nest site selection using a treatment-control approach. We hope to fill information gaps absent from recent research efforts in upland hardwood forests managed with prescribed fire and at landscape-scales rarely addressed in the current literature.

### Study Area

**Fig 2:**
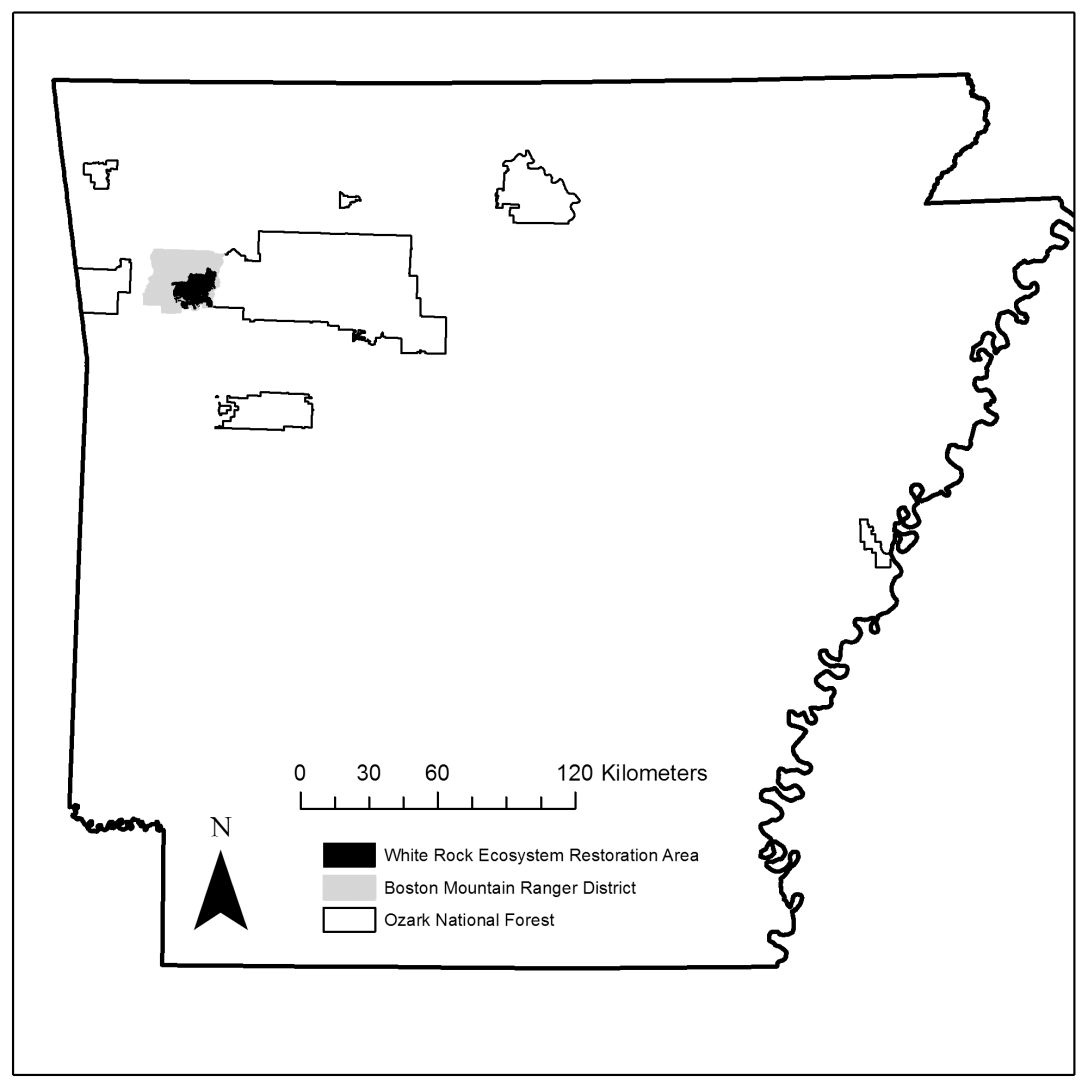
The White Rock Ecosystem Restoration Area is located in the U.S. Forest Service Boston Mountain Ranger District of the Ozark National Forest, Arkansas, USA.

The White Rock Ecosystem Restoration Area (WRERA) consists of 16,380 ha of prescribed fire treatment units composed of approximately 70% upland hardwood and 30% pine (*Pinus* spp.) or mixed-pine ecosystems. The WRERA is surrounded by untreated areas of similar composition and management, except for the use of prescribed fire, in northwest Arkansas, United States of America (USA, Fig 2). It is part of the main division of the Boston Mountain Ranger District (41,400 ha) of the Ozark-St. Francis National Forest. The WRERA is considered a high priority woodland and savanna restoration area for the USDA Forest Service managed under the CFLRP. Historically, woodlands and savannas maintained by frequent fire disturbances dominated the WRERA [13–15]. After fire suppression during the 20th century, the WRERA became dominated by closed-canopy hardwood forests of various oak (*Quercus* spp.) and hickory (*Carya* spp.) species. Understories consisted of canopy species regeneration, blackgum (*Nyssa sylvatica*), flowering dogwood (*Cornus florida*), Carolina buckthorne (*Rhamnus caroliniana*), blackberry (*Rubus* spp.) and devil’s walking stick (*Aralia spinosa*) [16]. Pine forest canopies were dominated by shortleaf pine (*Pinus echinata*) while understories consisted of hardwood and pine sprouts/seedlings. In 2002, managers at the WRERA implemented landscape-scale (>1,000 ha) prescribed fire designed to mimic historic fire regimes and to restore historic woodland and savanna conditions. Current management prescription for WRERA from the Ozark-St. Francis National Forest, Forest Plan describes woodlands as having “open canopies, sparse mid-stories and well-developed understories that are typically dominated by grasses and forbs, but also may become shrubby between fires and have a significant woody component” with 40 to 60% canopy closure [17]. Further, the prescription describes the management techniques to achieve these objectives including mechanical canopy removal, herbicide, and/or fire treatments. Note that since 2002, less than 5% of the restoration area has received either mechanical or herbicide treatments. Sixteen prescribed fire treatment units averaging 1,026 ha and ranging from 467 to 1,670 ha in size occurred on the WRERA. These units were typically a mixture of different stand types but were dominated by oak-hickory stands and were separated by constructed fire breaks and roads. Managers implemented prescribed fire from mid-March to early-April on a 3–5 year rotation in the 16 prescribed burn units, with units receiving a range of 1–4 prescribed fire treatments since 2002. On the WRERA, managers implemented prescribed fires with a combination of mostly aerial ignition supplemented by some ground ignition. During most prescribed fires, and usually because of implementation technique, fire treatments burned more than 75% of a treatment unit’s area. Managers would typically target 4 prescribed burn units annually, but due to variable weather, available personnel, and unit size, they would implement fire on total areas between 1000 and 5000 ha annually.

## Methods

We trapped wild turkey in and around the WRERA (35.6594, -93.9162 Lat/Lon) using rocket nets [18] from January to late-March 2012 to 2013. We captured all wild turkeys with the assistance and permission of the Arkansas Game and Fish Commission on the Boston Mountain Ranger District of the Ozark-St. Francis National Forest who also granted permission and access for our trapping efforts. Our trapping and research efforts also did not involve any endangered or protected species, and we made special effort to not affect any non-target species during our research. We marked all captured wild turkeys with an aluminum leg band and hens were fitted with a 110 gram Platform Transmitting Terminal (PTT) with Global Positioning System (GPS) capabilities and Very High Frequency (VHF) radio transmitter (North Star Science and Technology LLC, King George, Virginia) attached using a back-pack harness [19]. The PTTs recorded GPS locations 4 times per day during the 2012 nesting seasons and 8 times per day during the 2013 nesting season. GPS location accuracy was approximately 17 m and the GPS acquisition rate, the percentage of schedule location times when a location was actually recorded, was greater than 50% for all PTTs. Our PTTs transmitted GPS locations via the ARGOS satellite system (CLS America) every 120 hrs during 2012, and every 48 hrs during 2013. We determined nest-sites when >3 consecutive GPS locations were <20 m of each other or hen movement patterns exhibited nesting behavior. We visited each nest in the field using the GPS location and then determined nest-sites to <50 m using the VHF transmitters. We used GPS locations and VHF to monitor nest until abandonment or hatch. After abandonment or hatch we visited and searched for each nest bowl and only recorded vegetation measurements at nest-sites where we located the nest bowl. We recorded all nest-site vegetation measurements <48 hrs from the time the nest fate was determined.

We recorded 12 ground-collected habitat variables and 3 habitat variables derived from a Geographic Information System (GIS). We collected vegetation measurements using a 10-m radius circular plot centered at each nest-site. We also collected 155 random vegetation samples in circular plots based on a stratified random sampling design across the study area. We used a stratified random sampling design to ensure we adequately sampled vegetation in all possible prescribed fire implementation scenarios on the WRERA and unburned areas around the WRERA. At each circular plot, we recorded visual concealment at 0 to 1 m and 1 to 2 m in height [20], average percent ground cover type from four observations of a 1-m quadrat located 1 m from the plot center in the four cardinal directions [21], counts of live stems in diameter at breast height (dbh) classes along four 10-m transects from the plot center in all four cardinal directions, understory height at the plot center (m), canopy cover [22], slope (%), and slope position (% from bottom). Counts of live stems classes were trees 0–15 cm, 16–30 cm and >30 cm dbh and shrub classes ≤5 cm and >5 cm ground diameter. Our GIS-based habitat variables consisted of years since prescribed fire, distance (m) to nearest road, and USDA Forest Service cover type. We tested combinations of all variables for correlation and collinearity and when variables were correlated or exhibited collinearity, we selected the most biologically relevant variable for our analyses.

We calculated summary reproductive statistics to compare to previous wild turkey reproduction studies. Initial nesting rate (%) was the number of hens that attempted a first nest/total number of hens alive on March 1*100). Initial nest success (%) was the number of initial nest attempts that hatched ≥1 egg divided by the total number of initial nests*100. We classified a nest as hatched when at least one eggshell exhibited visual signs of pipping and both portions of the egg were intact. When we could not use eggshells to determine the fate of the nest, we located the hen within one week to verify the presence or absence of a brood. The renesting rate (%) was the number of second/third nests attempted by those hens that were unsuccessful in their initial attempt divided by the total number of hens that were unsuccessful in their initial attempt. The renesting success (%) was the number of renest attempts that successfully hatched ≥1 egg divided by the total number of renesting attempts. We calculated these statistics across the duration of the study, annually, and for treated and untreated units.

To test for differences in nest-site characteristics between successful and unsuccessful nests, we developed a set of six models of characteristics previously found to be important in the nest-site selection process of wild turkey [23–25]. These models consistent of nest fate (successful vs. unsuccessful) as the dependent variable and multiple management and vegetation characteristics at each nest-site as the independent variables. Our first three models compared the importance of visual concealment between successful and unsuccessful nest attempts. The first model (Visual Concealment Model) consisted of an intercept and three variables including: percent visual concealment from 0–1 m in height, percent visual concealment from 1–2 m in height, and the average understory height. Our second model (Stem Densities Model) compared selection based on live stem densities. The model consisted of an intercept and counts of stem densities from five size classes. Our third model (Ground Cover Model) compared the selection of percent ground cover classes. The model consisted of an intercept and variables for the percent grass, woody, and herbaceous ground cover classes. Our fourth model (Terrain Model) compared the selection of nest-sites based on terrain variables. The model consisted of an intercept and variables for percent slope, percent slope position from the bottom of the slope, and the distance to the nearest road. Our fifth model (Canopy Model) compared nest-site selection based on the openness of the canopy layer. The model consisted of an intercept and a variable for the percent of open canopy. We also fit a model (Fire Model) with prescribed fire treatment as a binary variable to test whether successful nests were placed in prescribed fire treatment areas or not in treatment areas. We compared these models for performance against an intercept only model using Akaike Information Criteria corrected for small sample size (AIC_c_) [26] to determine if habitat differences existed between successful and unsuccessful nest sites.

We next fit the previous five habitat models except the Fire Model to nest-sites and available locations in treated and untreated units to determine if the selection of nest sites over available sites differed between treated and untreated units (Table 1). We modeled nest-site selection using two data sets: one for our treatment group consisting of 31 nest-sites and 31 randomly selected available sites located in treated units and another for our control group consisting of 18 nest-sites and 18 randomly selected available sites located in untreated units. The randomly selected sample was selected from a stratified random sample of vegetation plots designed to cover all treatment and control units within the study area; the number of nest plots was not determined until all radio tagged turkeys nested and those nests were located. We compared all nest-site selection models to an intercept only model using AIC_c_ to test model performance, and all of our nest-site selection models performed better than the intercept only model in both treated and untreated units. We only report the results from models that had parameter estimates for main effects that were significantly different than zero. We did not incorporate cover type in our nest-site selection models as managers apply treatments in the same manner regardless of cover type.

**Table 1:** Comparison of eastern wild turkey (*Meleagris gallopavo silvestris*) reproductive parameters from across the sub-species' North American Range.

Study	Location	Years^1^	N^2^	INR^3^	INS^4^	RNR^5^	RNS^6^	Ecosystem^7^	Fire^8^
Kiss 2015 [36]	Manitoba, CAN	2	43	91	19	80	42	O	N
Kilburg et al. 2014 [10]	North Carolina, USA	2	65		33			P	Y (GS)
Little et al. 2014 [11]	Georgia, USA	3	82	70	42.1	36.8	42.9	P	Y (GS)
Pollentier et al. 2014 [37]	Wisconsin, USA	2	133	75.9	23.5^d^	32.4	43.1^d^	P/H/O	N
Byrne & Chamberlain 2013 [25]	Louisiana, USA	6	50	60	39.3	26.7	25	BH	N
Ludwig 2012 [38]	Delaware, USA	2	76		24.6			P	N
Williams 2012 [9]	Georgia, USA	2	62	71	41	62	40	P	Y (GS)
Moore et al. 2010 [39]	South Carolina	4	69	52.2	26.5			P	Y
Wilson et al. 2005 [40]	Louisiana, USA	4	24	33	38			BH	N
Nguyen et al. 2003 [29]	Ontario, CAN	2	22	68.2^c^	46.7^c^			UH/O	
Lehman et al. 2002^a^ [41]	South Dakota, USA	2	23/21^b^		54.7			O	
Miller et al. 1998 [35]	Mississippi, USA	13		72	27	34	25	P/BH	Y
Paisley et al. 1998 [42]	Wisconsin, USA	2	164	97.6	16	59.6	22.7	UH/O	
Badyaev et al. 1996 [27]	Arkansas, USA	2	81	88	21	50	3.5	UH	N
Thogmartin 1996^c^ [28]	Arkansas, USA	4	118	65	18	35	4.5	P	Y
Palmer et al. 1993 [43]	Mississippi, USA	8	143	72.7	30.8	34.8	26.1	P/BH	
Current Study	Arkansas, USA	2	65	92	26.5	37.5	7	UH	Y (GS)
Prescribed fire					14.6	20	0		
No Prescribed fire					31.6	100	7.7		

^1^Years = number of breeding season the reproductive parameters are based on

^2^N = number of wild turkey hens that the reproductive parameters are based on

^3^INR = Initial Nesting Rate (No. of first nest attempts/No. of hens available to nest; %)

^4^INS = Initial Nest Success (No. of initial nests to hatch ≥1 egg/Total No. of initial nest attempts; %)

^5^RNR = Renesting Rate (No. of Renest attempts/No. of hens who were unsuccessful in their initial nest attempt; %)

^6^RNS = Renesting Success (No. of renests to hatch ≥1 egg/Total No. of renest attempts; %)

^7^Codes denote the dominate canopy type in the study area: P = Pine, UH = Upland Hardwood, BH = Bottomland Hardwood, and O = Grassland/Agriculture

^8^Y = Prescribed fire was applied to the study area, N = No prescribed fire was applied to the study area, (GS) = At least some prescribed fire was implemented during the growing season

^a^Combined sample of Eastern and Rio Grande wild turkey

^b^Eastern wild turkey/Rio Grande wild turkey

^c^Adult hens only

^d^Product of daily survival rates

## Results

We captured 67 wild turkey hens of which 65 survived to the breeding season (30 in 2012, 35 in 2013). Of the 65 hens available at the beginning of the breeding season, 92% (60) attempted a first nest, and six of 28 were successful in 2012, and six of 32 were successful in 2013. Of the 48 hens that were unsuccessful in their first nest attempt, 35.7% (18) attempted to renest, resulting in 18 second nests and two third nests (Table 1). We documented an average nest incubation date of April 15 in 2012 and May 3 in 2013 (Table 2). Although prescribed fires occurred annually the latest prescribed fire in any fire season since 2002 on the WRERA occurred on April 2. Although multiple prescribed fires occurred during each year of our study, we did not document any nests destroyed by fire. We documented an average clutch size of 10.7 eggs (n = 16, SE = 0.84) and for successful nests we observed a 93.7% egg hatch rate. Nest success for initial nest attempts was 20% over the duration of the study (21% in 2012, 18% in 2013). Renest success rate was 5% over the duration of the study (1 of 20 attempts), 12.5% in 2012 (1 of 8 attempts), 0% in 2013 (0 of 12 attempts). Initial nest success for nests located in prescribed fire treatment units was 14.6% (6 of 41 attempts) and 31.6% (6 of 19 attempts) in untreated units, two nests were excluded since assignment to a treatment group was not possible (Table 1). We found that of all turkey hens that were unsuccessful and attempted a renest, none placed their renest attempt in a different treatment type from their initial nest attempt. We documented 7 renest attempts by 35 hens (20%) that were unsuccessful in their initial nest attempts in prescribed fire treated units, while we documented 13 renest attempts by 13 hens (100%) that were unsuccessful in their initial nest attempts in untreated units (Table 1). Renesting success was 0% (0 of 7 attempts) in prescribed fire treated units and 7.7% (1 of 13 attempts) in untreated units (Table 1).

**Table 2:** Comparison of eastern wild turkey (*Meleagris gallopavo silvestris*) incubation dates from across the sub-species' North American Range.

Study	Location	Year	Mean Incubation Date	N
Current Study	Arkansas (Ozarks)	2012	15-Apr	22
	Arkansas (Ozarks)	2013	3-May	27
Badyaev 1996 [27]	Arkansas (Ozarks)	1993	15-Apr	28
Thogmartin 1996 [28]	Arkansas (Ouachitas)	1993	19-May	11
	Arknasas (Ouachitas)	1994	4-May	18
	Arknasas (Ouachitas)	1995	23-Apr	15
	Arkansas (Ouachitas)	1996	13-May	14
			**Median Incubation Dates**	
Little et al. 2014 [11]	Georgia (JC)	2011	13-Apr	7
	Georgia (SL)	2011	22-Apr	8
	Georgia (JC)	2012	19-Apr	19
	Georgia (SL)	2012	21-Apr	25
	Georgia (JC)	2013	19-Apr	23
Vangilder et al. 1987 [44]	Missouri	1981	30-Apr	15
	Missouri	1982	28-Apr	10
	Missouri	1984	26-May	12
	Missouri	1985	4-May	36
Nguyen et al. 2003 [29]	Ontario	1999	3-Jun	22^1^
	Ontario	2000	17-May	
Fuller et al. 2013 [45]	New York	1996	11-May	6
	New York	1997	6-May	23

^1^Total number of radio marked hens from 1999 to 2000

When we compared differences in habitat characteristics between successful (n = 11) and unsuccessful (n = 38) nests across our six habitat models, no candidate model performed better than the intercept only model. Our comparison of nest site selection between treated and untreated units did indicate that differences in the selection of nest sites over available sites exist between treated and untreated units (Table 3). In our Visual Concealment Model, we found percent visual concealment from 0 to 1 m in height greater at nest sites than available sites in both treated and untreated units (Table 3). We also found that percent visual concealment from 1 to 2 m in height was significantly lower at nest sites than available in treated units but not in untreated units (Table 3). Our Stem Densities Model indicated the count of large trees was lower at nest sites than available sites and this relationship was only significant in treated units (Table 3). We also found that the count of small shrubs was significantly lower at nest-sites compared to available sites in treated units but not in untreated units, and the count of large shrubs was greater at nest-sites than available sites in untreated units but not in treated units. In our Ground Cover Model, we found the percent ground cover of woody vegetation was greater at nest sites than available sites in both treated and untreated units (Table 3). Our Terrain and Canopy models both did not indicate significant differences between nest-sites and available sites nor differences between treated and untreated units. We did not fit our Fire Model to the treated and untreated data sets because whether a nest was located within a prescribed fire unit versus not in a prescribed fire unit was used to differentiate between the two data sets and we would not have been able to estimate parameters associated with the prescribed fire variable.

**Table 3:** Model parameter estimates from five nest habitat models comparing nest-sites and available locations from units treated with early growing season prescribed fire (n(nest,available) = (31,31)) compared to units not treated with early growing season prescribed fire (n(nest,available) = (18,18)). P-values are presented for parameter estimates that were significantly different than zero based on a Wald's t-test. Diameter at Breast Height is denoted by DBH. Only Models with significant parameter estimates are reported.

	Treated			Untreated		
Model/Parameters	β	SE	p-value < 0.05	β	SE	p-value < 0.05
**Visual Concealment Model**						
% Visual Concealment (0–1 m)	3.72	1.4	0.008	7.25	1.8	0.006
% Visual Concealment (1–2 m)	-4.42	1.6	0.008	-4.22	2.9	
Understory Height (m)	1.08	1.06		1.35	1.1	
**Stem Densities Model**						
Small Shrubs (<5 cm ground diameter)	-0.09	0.03	0.009	0.01	0.03	
Large Shrubs (≥5 cm ground diameter)	0.04	0.05		0.21	0.11	0.05
Small Trees (<10 cm DBH)	0.19	0.1		-0.18	0.11	
Medium Trees (10–20 cm DBH)	-0.11	0.13		-0.17	0.14	
Large Trees (>20 cm DBH)	-0.41	0.13	0.002	0.02	0.14	
**Ground Cover Model**						
% Grass Ground Cover	0.06	0.04		0.04	0.04	
% Woody Ground Cover	0.05	0.01	< 0.001	0.03	0.02	0.049
% Herbaceous Ground Cover	-0.04	0.04		-0.09	0.07	

## Discussion

The CFLRP of the USDA Forest Service is not only a well-funded ($40 million annually) national program [12], but it marks a significant commitment to a forest management philosophy that focuses on the landscape-scale as opposed to previous management philosophies implemented at smaller scales. With this commitment comes a significant need for research on the effects of management practices on the scale of current implementation. Our study is a first attempt to address landscape-scale forest management, more specifically prescribed fire, in upland-hardwood dominated ecosystems and the effects on wild turkey. Our findings indicate that prescribed fire implemented on a landscape-scale (>10 000 ha) does not significantly improve nest survival or success of wild turkey in upland hardwood forests. We also documented initial nest success in prescribed fire treatment units lower than those documented in any other ecosystem similarly managed with prescribed fire (Table 1). This being the case, two major differences exist between our study and others: the implementation of early-growing season prescribed fire in an upland hardwood dominated ecosystem versus a pine dominated ecosystem and the scale of prescribed fire implementation (our average burn unit size ≥1000 ha versus a maximum of 240 ha [11]).

The relationship between prescribed fire and the nesting ecology of wild turkey or any ground-nesting bird is complex but likely composed of three critical components: exposure to prescribed fire, the changes to habitat due to prescribed fire, and the reproductive success of individuals in prescribed fire managed landscapes. The first critical component is the exposure of wild turkey nests to prescribed fire events. In some recent studies [9–11], researchers found that direct destruction of nests minimally affects turkey productivity. We too found no direct destruction of turkey nests by fire events during our study because fire events occurred before the majority of nest initiation. However, based on the current prescribed fire temporal implementation, wild turkey exposure to prescribed fire during both the periods preceding egg laying and incubation varies annually (Table 2). Wild turkey in our study on average began incubating nests on April 15 in 2012 and May 3 in 2013 (Table 2), a difference of 18 days. Based on our documented range of 18 days, in some years when nesting activity is earlier than what we documented, wild turkey exposure to prescribed fire events could be a concern. Annual variability in peak incubation is not something unique to our study as research in Arkansas [27], Missouri [28], and Ontario [29] documented annual variation of up to two weeks in average incubation dates. Such variation in incubation dates was found for this sub-species at other study areas along the western edge of the sub-species’ range but not further east (Table 2). These regional differences in annual variability of peak incubation may mean that the exposure of nesting wild turkey to prescribed fire may not only vary annually in some areas such as Arkansas, but may also vary across the sub-species’ geographic range where growing season prescribed fire is implemented.

**Fig 3:**
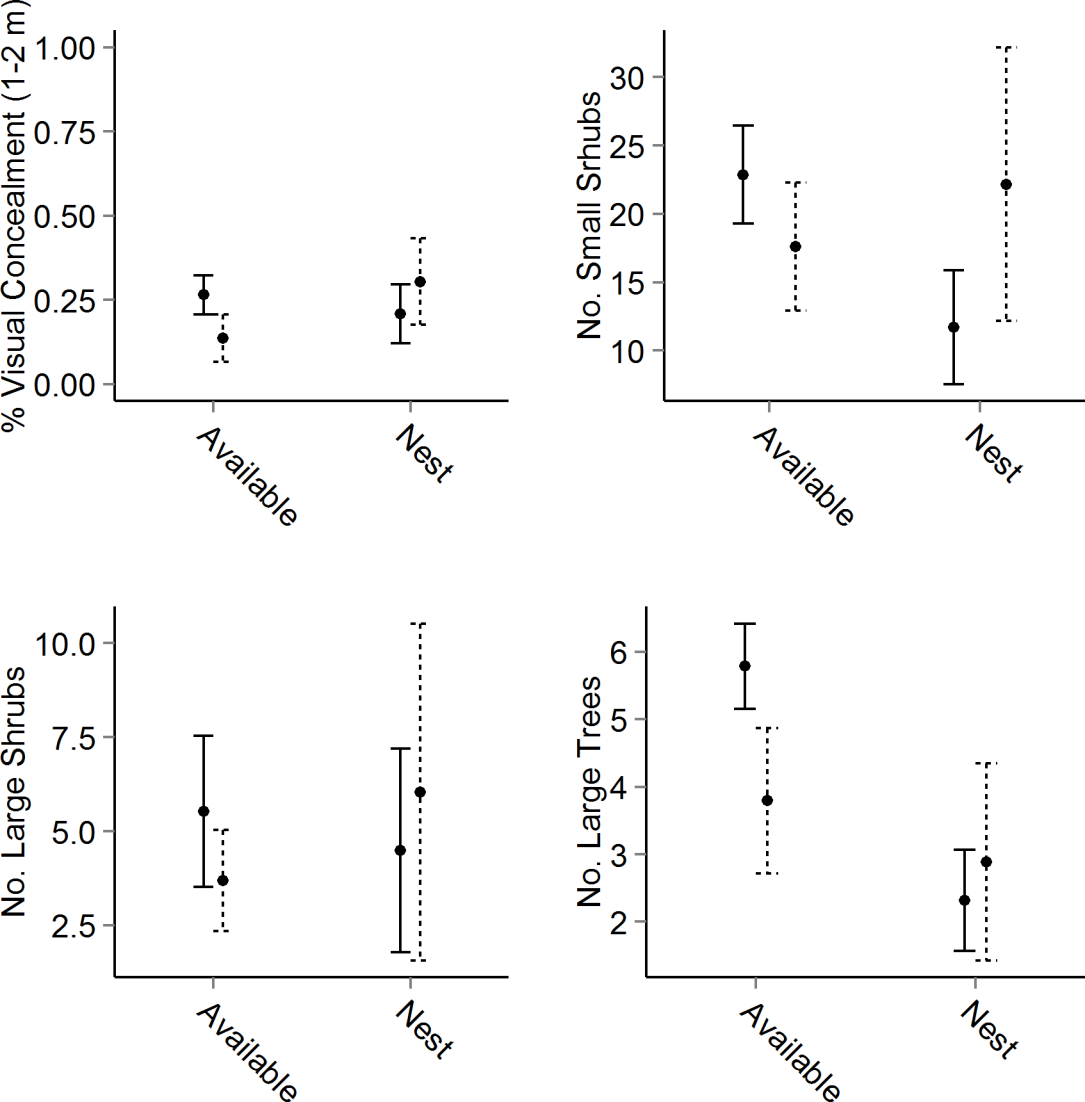
Mean (± 95% CI) for habitat variables collected at nest-sites and available sites in units treated with short-rotation prescribed fire (solid lines) and units not treated (dashed) at the White Rock Ecosystem Restoration Area, U.S. Forest Service Boston Mountain Ranger District of the Ozark National Forest, Arkansas, USA.

As part of the second critical component we examined how habitat characteristics used in nest site selection differed in treated and untreated units. In general, we found that nest site selection in treated and untreated units was different (Table 3). We observed that the variability in important nest site selection characteristics was less in treated than in untreated units (Fig 3). So it seems that prescribed fire treatments have reduced the variability or the range of available habitat characteristics used in selecting nest sites across the landscape (i.e., in both selected habitat and available habitat). Prescribed fire is typically associated with creating more diverse habitat and more habitat variability when implemented in similar terrain and ecosystems as opposed to our findings [30]. We also observed differences between nest site selection in treated and untreated units in our stem density model (Table 3). The selection of nest-sites based on there being fewer large trees and small shrubs was significant in treated units but not in untreated. We expected to observe these differences because prescribed fire can increase canopy tree mortality and reduce woody shrubs [30–32]. Our ground cover model indicated wild turkey select nest-sites similarly based on ground cover in treated compared to untreated units (Table 3). In both treated and untreated units wild turkey selected for nest-sites with more woody stem ground cover which was typically made up of woody vines.

The third critical component and most informative of beneficial or deleterious effects on the population is the ability of individuals to successfully reproduce in prescribed fire managed landscapes. This component is composed of the effects of the previous two components and the direct effects habitat may have on nest success. Other studies of nesting ecology [24,33–35] all found that habitat characteristics around nest sites have a significant influence on survival. Unlike the results from previous studies though, we did not find any habitat models or variables that predicted successful nests. Although our total number of nests tracked was comparable to other studies, the lack of successful nests limited our ability to characterize the habitat differences between unsuccessful and successful nests. Nest success was higher in untreated units compared to treated units. When we compare the nest success rate for prescribed fire treated units to those of other studies in ecosystems managed with prescribed fire we find that our 14.6% nest success is the lowest documented in a study area managed with prescribed fire (Table 1). We further documented a similar renesting rate to many other studies of wild turkey, but our documented renest success was among the lowest reported and only comparable to rates previously documented in the Arkansas Ozarks (3.5%) [27] and the Arkansas Ouachita Mountains (4.5%) [24]. Our low renesting success rates are of concern because Williams [9] found that destruction of wild turkey nests by growing season prescribed fire requires a substantial renesting effort and nest success to buffer wild turkey populations against significant declines.

Our study addresses three critical components of the relationship of wild turkey reproductive ecology and prescribed fire in upland hardwood dominated ecosystems. Our findings indicated that prescribed fire has changed the nest site and available habitat characteristics on the WRERA compared to untreated areas. One important finding from our study was that no wild turkey nests were directly destroyed by prescribed fire. The combination of the timing of prescribed fire events and the average incubation dates suggests the exposure of incubating wild turkey to prescribed fire is minimal. Our documentation of nest sites in units managed with prescribed fire indicated nest success was approximately 50% lower in those units compared to nest sites located in unmanaged units. The mechanism behind this difference is still unclear as successful and unsuccessful nest sites did not differ in the habitat characteristics we measured. In conclusion, we found no evidence that wild turkey have benefited from the implementation of short-rotation early-growing season prescribed fire on the landscape-scale and further study is needed to determine if prescribed fire may in fact be contributing to observed wild turkey declines on the WRERA.

## Supporting Information

S1 Dataset Nest success and site selection data sets. (XLSX)
